# Consensus-based recommendations for strengthening emergency care at primary health care level: a Delphi study

**DOI:** 10.1080/16549716.2022.2156114

**Published:** 2023-01-05

**Authors:** Meghan Botes, Judith Bruce, Richard Cooke

**Affiliations:** a Department of nursing education, University of the Witwatersrand, Parktown, South Africa; b Department of Family Medicine and Primary Care, University of the Witwatersrand, Parktown, South Africa

**Keywords:** Enhancing emergency care, entry level facilities, frontline care, recommendations, consensus

## Abstract

**Background:**

Emergency care at a primary health care (PHC) level must be strengthened to reduce overall mortality and morbidity in any country. Developing recommendations for improvement in this area should take into consideration the context and nuances of the current emergency care system and primary health care context. Contribution to policy from the experts in the cross-cutting fields of PHC and emergency care is lacking.

**Objectives:**

This study aims to evaluate the strengths and weaknesses of emergency care in primary health settings and develop consensus-based recommendations for the strengthening of emergency care at this level.

**Methods:**

Using a modified Delphi technique, data were collected from various data sources to evaluate the strengths and weaknesses of emergency care at PHC level, from which recommendation statements were developed. These recommendations were proposed to a panel of experts using a Delphi survey to build consensus on 14 recommendations to strengthen emergency care at PHC level.

**Results:**

Ten experts were recruited to participate (n = 10) with a response rate of 90% in round II and 80% in round III of Delphi. Recommendations broadly addressed the areas of education and training in emergency care, the role and placement of various actors, leadership in emergency care and the development of a national plan for emergency care. Consensus was reached in round II for 97.61% of the statements and after modification based on open-ended comments, 98.21% consensus was reached in round III.

**Conclusion:**

Strengthening emergency care at primary and subsequent levels of health care requires a coordinated effort and mandate from authority in order to effect real change.

## Introduction

The integration of effective emergency care into primary health care (PHC) directly reduces mortality and disability, and maximises impact across the health system as a whole [1,2]. Globally, there has been neglect in the area of emergency services offered in PHC settings, particularly in low- and middle-income countries (LIMC) [3]. Various factors have led to this neglect, such as the perception that emergency care is a costly service, poor resource availability in LMICs, and the lack of data to motivate for improved emergency care [4].

Within PHC settings, emergency care is not considered to be a major part of the services offered with the focus mainly on the prevention of disease and promotion of health. An effective emergency care system requires a strong network of support and a clear pathway from presentation to treatment [5]. The South African PHC system has weaknesses in the provisioning of emergency care relating to skills capacity and a dysfunctional referral system [6–8]. Specialised emergency skills and resources required to deliver this type of care are often not found in PHC contexts, bringing into question the quality of emergency care rendered at this level. Poor resourcing may be attributed to a historically hospicentric approach that siphons resources to deal with complicated health problems not treated at PHC facilities. Emergency care at PHC level is situated within a broader system of care.

While the broader system of PHC is well defined, the availability of resources, consultative support and the effectiveness of the referral system specifically related to emergency care is lacking. These aspects of any health service are usually defined in the relevant health policy that is responsible for giving direction to a health care system and therefore has a direct effect on the services offered. Health policy reflects the priorities of a health care system or service, and the processes involved in its development. Emergency care has been mandated by the World Health Organisation (WHO) as a national responsibility and therefore this priority should be reflected in a country’s health policy [9]. Internationally, various strategies have been employed for the improvement of emergency care in different countries [5]. It is important that strategies for improvement are contextually relevant and functionally integrated into the existing system of care. This can be done effectively only if the process is informed by a rich understanding of the broader context and the reality of the current state of emergency care in PHC settings.

## Aim

The aim of the study was to evaluate the strengths and weaknesses of emergency care in primary health settings and develop consensus-based recommendations for the strengthening of emergency care at this level.

## Methods

A modified Delphi study was conducted to develop consensus among a group of experts regarding recommendations to improve emergency care at primary health care level. Data were collected and triangulated from multiple sources to develop evaluative conclusions about emergency care in the PHC system. The evaluative conclusions formed the basis of recommendation statements for strengthening emergency care for which consensus was sought from a panel of experts in the cross-cutting fields of emergency care, primary health care and policy development.

### Delphi survey – round I

The purpose of the first round of Delphi was to formulate recommendation statements based on triangulated data from multiple data sources.

### Sample and sampling method

Round I involved the collection and analysis of data from various data sources to describe the context, content, process, and actors involved in emergency care at PHC level and to formulate strategic recommendation statements.

#### Data source 1

Random selection of facilities with maximum variation sampling technique for participant recruitment yielded a sample of 22 nurses and doctors working in PHC facilities of various levels across the Gauteng province.

#### Data source 2

The policies selected for content analysis suggested as key for giving directive for the district health care services were identified from participants during interviews and further documents identified using a snowballing technique.

#### Data source 3

Key informants were defined as either experts in the cross-cutting fields of practice, namely emergency care and primary health care or involved in the policy development process of the policies of focus using the following criteria:

- Involvement in policy development or implementation in either of the cross-cutting fields at a national, provincial, or district level
- Academic Heads of Departments in academic institutions offering programmes in any of the cross-cutting fields
- Management of clinical departments of any of the cross-cutting fields at PHC level.

Using a network sampling approach (also known as the snowballing method), academic and clinical heads of various departments, as well as representatives from the national office of emergency services, were recruited along with stakeholders involved in the development of some of the policies of focus.

### Data collection

Three data sources were used to collect the data for round I Individual, semi-structured interviews were conducted with participants about their experiences in managing emergencies at PHC level. The policies of focus were critically analysed and described. The relationship between the various directive legislations, the National Health Act 61 of 2003, as well as the guidelines and policies were described. Interviews with key informants provided insight on policy development and implementation with a specific focus on emergency care in the PHC system.

### Data analysis

Data from round I were analysed and triangulated using qualitative content analysis. Evaluative conclusions were deductively formulated. These formed the basis of the 14 recommendation statements presented in rounds II and III of the Delphi study.

### Delphi survey – rounds II and III

The purpose of rounds II and III of Delphi was to build consensus between a panel of experts regarding the proposed recommendation for strengthening emergency care at a PHC level

### Sample and sampling method

Key informants (Data source 3) who participated in round I of the study were invited to participate in the Delphi survey. Ten participants were recruited (n = 10) with a response rate of 90% in the second round and 80% in the third round.

### Data collection

A survey tool including the 14 recommendation statements formulated in round I was developed. Participants in rounds II and III were required to rank their level of agreement of each statement on a 4-point Likert scale as either ‘Strongly agree’, ‘Agree’, ‘Disagree’ or ‘Strongly Disagree’. The survey was circulated via an online platform and data were stored on a secure online database. Reliability of the survey tool was measured using the Cronbach's alpha coefficient, which produced a reliability coefficient of 0.72 indicating good internal consistency of the Delphi scale.

### Data analysis

Consensus was set at 80% of the participants answering either ‘agree or strongly agree’ or ‘disagree or strongly disagree’ and was reported using measures of central tendency and distribution analysis. Open-ended sections were included for participants to comment on each recommendation. Comments were analysed using qualitative content analysis and integrated into changes presented in round III of the study. Open-ended comments were reviewed, coded per item and compared with the existing statement before incorporation.

## Ethical considerations

Ethical approval to conduct the research was granted the Human Research Ethics Committee (Medical) of the University of the Witwatersrand. Written informed consent was obtained for participating in the study and for digital recording of the interviews. Confidentiality and anonymity were maintained for participants and facilities through allocating pseudonyms to the participants and transcripts. An information letter informed participants of their choice to participate and option to withdraw at any stage of the research process. Rigour of the study was ensured by anonymity of participants, iteration through multiple rounds of consensus building, controlled feedback by including the option for participants to provide feedback and comment, and statistical consensus in the Delphi survey. The Delphi method contributed to the validity of findings by allowing experts in the field to interpret, contextualise and ensure relevance of the proposed recommendations. Triangulation of the results from round I contributed to the richness of the data and trustworthiness of the findings presented.

## Results

This section presents the findings from round II and III of the Delphi survey.

### Demographic characteristics of study participants

The majority of the sample were males 80% (n = 8), in the age bracket of 50–59 years (60%; n = 6) and were in possession of a medical degree as their basic professional qualification, indicative of the general demographic of health care management in South Africa being predominantly white male [10]. Half of the key informants (n = 5) have had between 10 and 20 years of experience in their current field, with 20% of participants (n = 2) retired and consulting in the Department of Health. All participants had postgraduate qualifications ranging from certificates to doctoral degrees (Table 1). The institutional memory and experiential knowledge shared by the participants has created a broad base upon which recommendations were formulated.

**Table 1.** Demographic characteristics of study participants.

Category	Frequency (n)	Percentage (%)	Category	Frequency (n)	Percentage (%)
Gender					
Female	2	20	Male	8	80
Race					
Black	4	40	White	4	40
Indian	1	10	Coloured	1	10
Age					
30–39	1	10	40–49		
50–59	6	60	60–69	1	10
70–79	1	10			
Undergraduate qualifications					
Bachelor of Medicine and Surgery	6	60	Bachelor of Nursing	1	10
Bachelor of Dentistry	1	10			
Post graduate Qualification					
Postgraduate Certificates/Diplomas	10	100	Master’s Degree	6	60
Doctoral Degree	2	20			
Number of years working in current field					
5–10 years	1	10	11–20 years	5	50
21–30 years	2	20	Currently retired	2	20

### Delphi survey findings

Consensus was reached for each of the recommendations on the Delphi Scale and the overall agreement level was 97.61% in round II. Open-ended comments provided details and suggestions for the modification of seven of the recommendations which were set in a second survey using the same scale and sent to participants in round III. There was an overall improvement in the consensus from round II (97.61%) to round III (98.21%). Nine of the 14 recommendations (64.28%) had increased mean scores in round III while others remained unchanged. The recommendations were classified under three broad categories. Table 2 depicts experts’ levels of agreement for each recommendation

**Table 2.** Experts’ level of consensus on recommendations to strengthen emergency care.

Final Recommendations		Consensus (%)
Education & Training	Basic emergency skills training is required for all health care practitioners working at District Health Disagree Care facilities.	100
	Emergency skills training should be context specific and appropriate for the scope and capacity of the health care practitioner.	100
Role Definition	A strategy for placement of specialised emergency care practitioners needs to be developed to include the District Health Care System as a setting for their practice.	88
	The role and definition of Emergency Care as an overarching system of care, and the role and definition of Emergency Medical Services and Emergency Medicine should be clarified.	100
Leadership in Emergency Care	A position of leadership for emergency care at a national health level is required to ensure that emergency care becomes a priority for the National Health Care System.	100
	One office for emergency care should be established which covers the spectrum of emergency care including emergency medical services to ensure an integrated approach to emergency care	100
	Leadership at a district level to champion the cause of emergency care and provide support for the implementation of policies is recommended, and therefore dedicated posts for emergency specialist practitioners should be created within the District Health System.	100
A national plan for Emergency Care	Policy requirements and standards should acknowledge variations in context between facilities and provide appropriate direction for these different levels of care.	100
	A multi-level emergency network or system that coordinates emergency care at all levels is needed.	88
	A clear referral process, appropriate for various contexts should be developed at the level of each district involving stakeholders from all facilities and coordinated by the emergency care office.	100
	A consolidation of emergency care centres should be conducted to identify facilities within the District Health Care System that have greater capacity to manage complicated emergencies, while lower level facilities are empowered to stabilise and refer.	100
	A National Emergency Care strategy must be developed in order to form a strong emergency system that integrates functionally with the District Health Care System.	100
	The content of a national emergency care strategy should emphasise the processes and quality of the service and not only the physical requirements, with a specific focus on the health outcomes of emergency care including mortality and morbidity.	100
	The development of a system for data capturing regarding emergency services is needed to provide feedback on the efficiency and effectiveness of the service, this includes the development of a diagnostic coding system.	100

## Discussion

The recommendations proposed cover focus on areas in need of improvement in the emergency care system. These broad areas include education and training, the role and placement of various actors, the need for leadership in emergency care and the need for a national strategy for emergency care.

### Education and training in emergency care

The expectation of any PHC facility from its most basic package of services to the district-level hospital, is that there is the capacity to stabilise any acutely ill or injured patient and refer to the appropriate next-level facility [6,11]. Emergency skills are part of the most basic health care educational curricula; however, the skills acquired are not retained in settings where emergency care is not the focal service offered [12]. Continuous educational drives that are context-specific and appropriate for the scope of practice of the health care practitioner, are thus required.

Like many LMICs, and in particular sub-Saharan Africa, nurses without postgraduate training make up the largest portion of the health workforce [13]. Nurses in primary health care and rural facilities practice amid serious resource-constraints and often have little or no formal training in emergency skills, and yet they are expected to manage acutely ill and injured patients independently [13,14]. This points to a need for nurses to be appropriately prepared for situations in settings that are very different to the ideal emergency department setting. In response to the call for contextually relevant emergency skills training, many countries have developed specific courses for practitioners in rural contexts.

In contrast to a once-off course, there is a need to develop a system of continuous education [15]. The retention of skills post-emergency training also depends on exposure to emergencies. Various strategies for rotation between facilities and opportunities for exposure have been piloted with some success, highlighting the need for a different approach [16]. The use of educational technologies such as simulation and blended learning approaches may assist in providing immersive experiences for health care professionals practicing in areas where exposure to emergencies is not sufficient to retain the skills required with the careful consideration of limited access to educational technology [17]. Training networks and partnerships may assist in knowledge and resource sharing without negating the context-specific needs of primary health care settings.

The gap between basic emergency skills and specialist practice is not clearly defined in the literature leaving practitioners uncertain of their scope the objectives of training difficult to define. Ideally, the mentorship and consultation of specialist emergency practitioners with general primary health care practitioners would assist in closing this gap, but due to the scarcity of specialist emergency practitioners this is not always possible. This highlights the need for an increase in the training of specialist emergency practitioners – particularly in the fields of emergency medicine and emergency nursing [18,19].

Models that have been trialled in other countries include the upskilling of various cadres of health care practitioners, also known as task shifting. Terry et al. describe the concept of task shifting as upskilling a health care practitioner beyond their usual scope of practice in order to meet the identified needs of the health care system [20]. Similarly, task sharing emphasises the more supportive multidisciplinary approach as opposed to complete autonomous shifting of skills [21]. This is proposed as potential solution, particularly for specialised skills involved in emergency care. This model requires a multi-stakeholder approach to ensure regulation of their new scope of practice and support for these practitioners. The implementation has yielded positive results in countries, such as Uganda where nurses were specifically trained in emergency care over two-year period and the growing cadre of professionals known as medical licentiates or clinical associates in countries, such as Zambia, South Africa and other sub-Saharan countries [22,23]. The mentorship, placement, remuneration and regulation of these professionals would need to be considered for each context with regard to the placement of various actors in the emergency care system.

### The role and placement of various actors

The District Health Care system is a platform for the delivery of PHC services, consisting mainly of generalist nurses and doctors. Speciality practice in this setting includes nurses who have acquired a postgraduate qualification in PHC and doctors who have specialised in the field of Family Medicine [24]. A District Specialist Team exists for the purpose of oversight for each district consisting of a ‘family physician, a primary health care nurse, an obstetrician and gynaecologist, an advanced midwife, a paediatrician and a paediatric nurse’ with the later addition of an anaesthetist for the oversight of medical emergencies and perioperative care [24]. The team is intended to be led by a family physician and a primary health care nurse as PHC is the focus of care.

Emergency care is a distinct field of medical and nursing practice with a different approach. The void in contribution from emergency care experts in this team is concerning. A study exploring the rural health care system of America proposed a model of partnership between rural health and emergency medicine with the goal of integrated system [25].

A national plan for emergency care should therefore include a strategy for the placement of specialist emergency nurses and doctors within the district health care system and for increasing the training of specialists in this field [19]. The skills set offered by these practitioners will improve health outcomes and decrease the burden of disease caused by acute illness and injury [26]. Included in this strategy should be the consideration for the upskilling and task shifting or sharing for PHC practitioners currently dealing with emergencies at this level of care.

An emergency care system is a network of service including emergency medical services whose focus is on pre-hospital care and inter-hospital transfer with the goal of getting a patient to an appropriate facility for care as well as disaster preparedness and response [27]. The extended scope of emergency medicine includes that of prehospital care and disaster management but has its focus largely on in-facility care [5]. In South Africa, the scope of these speciality fields has not been determined or recognised in health care policy. The term ‘Emergency Health Services’ is used broadly to refer emergency care in South African literature and policy documents [28]. The use of such vague terminology results in a lack of accountability as roles and responsibilities are not clearly defined. In order to coordinate a well-functioning system of emergency care, these terms and roles must be defined and acknowledged in policy. Currently, there exists a national office for emergency medical services, however the scope of this office is limited to the prehospital environment and disaster management, hence the recommendation to create an office, which encompasses the full spectrum of emergency care.

### Leadership in emergency care

A position of leadership for emergency care at the national health level is required to ensure that emergency care becomes a priority for the National Health Care System. Health care leadership with the ability to coordinate complex systems and empower people to achieve set goals, has become emphasised above basic managerial skills in health care management [29]. Without a champion for a cause, agenda setting for decision-making and policy development is left to the prerogative of the bureaucratic authority such as ministerial teams and government officials [30]. Clinicians need to be included in the process of agenda setting, and national leadership is needed to ensure integration with the national health care system goals and structures. This requires the national government to create a platform for leadership in emergency care to coordinate care and contribute effectively to decision-making. The development of a system of emergency care requires not only oversight and coordination but vision and championship.

Similarly, there exists the need for leadership at a district level to champion the cause of emergency care and provide support for the implementation of policies, and therefore dedicated posts for emergency specialist practitioners are needed within the District Health Care System. National leadership would require a team of people at different levels of the health care system in order to implement a multi-level strategy for emergency care, inclusive of primary-, secondary- and tertiary-level health care facilities.

An office of emergency care without the support of clinicians and clinical governance would be redundant and hold theoretical value without the capacity to extend its reach to the District Health facilities in need of support and mentorship in emergency care.

The role of emergency nurses as advanced practitioners in a PHC setting, has not yet been clearly explored [31,32]; however, their ability to offer a leadership and supportive role within the district may be a consideration, making them candidates for championing emergency care at district health care level [33].

A mandate from the national government is necessary to ensure that the office has the power to execute its responsibility and effect change [5,34]. Recommendations developed by various associations and representative bodies do not always reach the agenda setting table and therefore a mandate given by national authorities may increase the effectiveness of this office. The responsibility for the establishment of a functional emergency care system has been highlighted by the WHO as an essential role of government [5,35] founded on civilians’ right to access emergency care and as such, should be the key priority of any health care system [26]. This provides strong motivation for the establishment of a national emergency care office.

### A national plan for emergency care

A national plan for emergency care should include the development of appropriate policy to guide emergency care, the development of a national strategy for emergency care including the development of a clear referral system and network of emergency care services. For a policy to be effective, it must be contextually appropriate and relevant to the needs of the particular context it must function in [36]. Dynamic policy effectiveness suggests that policy should be able to adapt to changing circumstances and still give appropriate direction for the achievement of the policy goals [36]. Dynamic effectiveness also asserts that re-evaluation and periodic re-assessment are beneficial, as seen in Australia where a recent update of their emergency care framework proposed strategic improvement in the coordination of care guided by a national strategy or framework [37].

Contextual relevance considers factors such as resource availability, human resources for health, the political and economic environment and the socio-cultural context [38]. This will require collaborative efforts from various stakeholders at different levels of the health care system to ensure that different contexts are well represented, and a rich understanding of the needs is achieved. A policy that is developed from a broad base of understanding and insight is more effective than a top-down developed policy [38]. The coordination of this process would be a key function of the proposed national office for emergency care.

Strategies to develop or improve emergency care in various LMICs have proven their worth in the decrease in mortality and morbidity and ultimately the burden of disease caused by acute illness and injury [5]. The WHO proposes a framework for emergency care that allows interrelation between various levels of care and various stakeholders or service providers [39]. The WHO framework describes the process of emergency care from activation to definitive care and takes into account various entry points and the emergency care capacity at these varying levels of care supporting the recommendation for a multi-level emergency care plan [5]. The underpinning principle of improvement of the emergency care system is broad-based assessment in order for contextual application of the proposed framework. The Pacific region spanning across multiple countries undertook to complete the task of assessment and identification of strategies for improving emergency care in their own context. Their findings, similar to this study, highlighted the areas of training, emergency equipment and resources, the need for leadership, the need for process direction in the form of policy and the need for capturing and analysis of emergency care data [40]. This multi-national strategy brings together the necessary stakeholders and allows for a plan that considers every level of care.

Emergency care in a PHC setting will be automatically strengthened once a national system of emergency care is established to develop and support emergency care offered at this level. This development includes defining the geographical boundaries, identifying various levels of care based on the capacity of the facilities within a region, recruiting the contribution and involvement of stakeholders at each level, and finally, developing a network of support that links the various levels [41]. Effective emergency care is measured by its ability to move a patient rapidly and seamlessly through the system with the goal of bringing them to definitive care [5].

A well-coordinated system requires the development of an efficient referral system as an additional role of the emergency care office. The South African referral system is described as obstructive and a referral system that is coordinated at a systemic level but relevant to the local context is recommended [4].

The need to build a supportive network between facilities is vital and ideally, stakeholders from the various levels of facilities should be involved in the process of developing a referral pathway to ensure collaboration, ownership and accountability for the effectiveness of the system. The financing of collaborative efforts becomes tricky as the allocation of budgets are considered and competing agendas are highlighted. A national budget should therefore be identified for emergency care [4]. The development of a system for dispersal and optimal use of funds and resources is key, particularly with respect to improving emergency care in low-resourced settings [42].

Various models for the development of a network of facilities in the system of emergency care have been proposed with most countries employing a wheel and spoke approach. This approach has a ring of peripheral facilities referring to a central higher-level facility for definitive care. A nodal approach allows for various facilities to be interconnected for the sharing of resources, information and support. A combination of these models would allow for the supportive nature of a nodal approach and collaboration between facilities, while the goal of reaching a dedicated emergency care facility able to offer definitive care is still realised. The conceptualisation of such a system would require an assessment of the current emergency care system in order to develop a wide view of the current facilities and resources.

Assessment models such as the broad workshop-based assessment employed in the Pacific region, or the use of the WHO ECAT (emergency care assessment tool) as part of the process should be considered [5,43]. A national assessment would provide essential data needed to develop an effective strategy. An assessment of this nature would also provide data on resources and identify areas with increased capacity for emergency care and those in need of more support. Physical requirements for infrastructure, equipment, consumable and human resources are vital for effective care [4]. The processes, which detail all aspects of care and the clear delineation of various role players and networks, should be the focus of policy. To ensure quality of care, there must be a holistic and contextual approach to policy that gives appropriate direction and detailed processes for ease of implementation.

Monitoring and evaluation of the system of care and therefore the ability to effect quality improvement is largely dependent on the availability and quality of data [44]. There is an obvious dearth of data on emergency care outcomes, a challenge experienced by many LMIC’s [5]. The quality of the system cannot be evaluated and therefore the motivation for improvement is difficult to justify. The development of a system for data capturing regarding emergency care is needed to provide feedback on the efficiency and effectiveness of the service.

Indicators for monitoring must be clearly defined to ensure high-quality relevant data. A data system is needed that is simple, effective and can be applied in various contexts and levels of care. This will also allow for the development of an understanding of the contextual needs of different facilities and regions and focus policy, training and interventions appropriately. Information and technology support for record keeping and the availability of equipment for this purpose play a major role on the success of this system, and must be taken into consideration.

## Conclusion

A well-coordinated emergency care system with adequately trained health care providers, clear direction and leadership and a contextually relevant referral system should be a national priority. Fourteen consensus-based recommendations for strengthening emergency care at primary and subsequent levels of health care are presented, which if implemented have the potential to improve care, and reduce the burden of mortality and morbidity caused by poor emergency care beginning at the most basic, entry level of care to the most advanced facilities. The need to conduct a broad-based assessment of emergency care nationally has been highlighted in order to support the recommendations. There is an opportunity to build a strong chain of survival by linking and coordinating vital parts of the emergency care system in South Africa.

## Limitations

This study was a focused assessment in a particular geographical area within one province in South Africa, and therefore the findings might be generalisable only to similar study contexts. Due to geographical constraints, a limited number of participants may have restricted the perspectives to context-specific findings indicating the need for a national assessment and strategy.

## Paper context

Globally, emergency care at a primary health care (PHC) has been identified as an opportunity to reduce mortality and morbidity related to acute illness and injury, however, strategies to improve care at this level require a comprehensive assessment and a mandate from authority within policy to effect real change. This study aimed to assess emergency care at a PHC level and present consensus-based recommendations for strengthening emergency care in the PHC setting.
